# Cyclic Polypeptide D7 Protects Bone Marrow Mesenchymal Cells and Promotes Chondrogenesis during Osteonecrosis of the Femoral Head via Growth Differentiation Factor 15-Mediated Redox Signaling

**DOI:** 10.1155/2022/3182368

**Published:** 2022-03-03

**Authors:** Jiazheng Chen, Zichen Cui, Yi Wang, Linmao Lyu, Changgong Feng, Dianjie Feng, Yifan Cheng, Ziqing Li, Shui Sun

**Affiliations:** ^1^Department of Joint Surgery, Shandong Provincial Hospital, Cheeloo College of Medicine, Shandong University, Jinan, Shandong 250012, China; ^2^Department of Joint Surgery, Shandong Provincial Hospital Affiliated to Shandong First Medical University, Jinan, Shandong 250021, China; ^3^Orthopaedic Research Laboratory, Medical Science and Technology Innovation Center, Shandong First Medical University & Shandong Academy of Medical Sciences, Jinan, Shandong 250117, China; ^4^Department of Emergency Medicine, Qilu Hospital of Shandong University, Jinan, China

## Abstract

Osteonecrosis of the femoral head (ONFH) is a debilitating disease that is closely associated with the clinical application of high-dose glucocorticoids. Elevated oxidative stress contributes to the pathophysiological changes observed in ONFH. The lack of effective treatments besides surgical intervention highlights the importance of finding novel therapeutics. Our previous studies demonstrated that D7, a cyclic polypeptide, enhances the adhesion, expansion, and proliferation of bone marrow mesenchymal stem cells (BMSCs). Therefore, in this study, we investigated the therapeutic effects of D7 against ONFH in BMSCs and evaluated the underlying mechanisms. First, we screened for ONFH risk factors. Then, we applied D7 treatment to steroid-induced ONFH (SONFH) in an in vitro model produced by dexamethasone (DEX) to further elucidate the underlying mechanisms. We found negative correlations among oxidative stress marker expression, growth differentiation factor 15 (GDF15) levels, and ONFH. Furthermore, we demonstrated that DEX inhibited the proliferation and induced apoptosis of BMSCs by suppressing GDF15/AKT/mammalian target of rapamycin (mTOR) signaling. D7 alleviated DEX-induced BMSCs injury and restored the chondrogenic function of BMSCs by activating GDF15/AKT/mTOR signaling. In addition, DEX-induced excessive reactive oxygen species (ROS) generation was an upstream trigger of GDF15-mediated signaling, and D7 ameliorated this DEX-induced redox imbalance by restoring the expression of antioxidants, including superoxide dismutase (SOD) 1, SOD2, and catalase, via regulation of GDF15 expression. In conclusion, our findings revealed the potential therapeutic effects of D7 in SONFH and showed that this protective function may be mediated via inhibition of DEX-induced ROS and activation of GDF15/AKT/mTOR signaling, thereby providing insights into the potential applications of D7 in SONFH treatment.

## 1. Introduction

Osteonecrosis of the femoral head (ONFH) is a progressive disease that causes collapse of the joint cartilage and the femoral head, leading to substantial hip pain and loss of joint function [1, 2]. Clinical application of glucocorticoids (GCs) is the leading cause of ONFH, also called steroid-induced ONFH (SONFH). The pathophysiological changes associated with SONFH include disruption of bone marrow-derived mesenchymal stem cells (BMSCs) self-renewal; acceleration of an imbalance among osteogenic, chondrogenic, and adipogenic differentiation [3]; circulatory impairment [4]; modified artery constriction [5]; and intramedullary pressure changes [6]. To date, numerous surgical procedures have been applied for ONFH treatment, including total hip arthroplasty (THA) and joint-preserving surgeries, such as core decompression (CD), osteotomy, and free vascularized fibular grafting. Additionally, CD combined with cell transplantation prevents early-stage ONFH from femoral head collapse and delays the need for THA [7]. Although these treatments can substantially improve the quality of life of patients, postoperative complications, such as infection, deep vein thromboembolism, peroneal nerve palsy, proximal femoral fracture, ankle functional impairment, dislocation, periprosthetic fracture, and prosthesis loosening, cannot be neglected [8–10]. Therefore, it is essential to identify novel therapeutic targets in early-stage ONFH to prevent further pathophysiological changes and to delay surgical interventions.

Reactive oxygen species (ROS) contribute to the pathogenesis of SONFH [11]. Under normal physiological conditions, ROS are produced by cell metabolism. A balanced level of ROS plays a significant role in cell homeostasis and signal transduction [12]. However, under stress conditions, the balance between the formation and degradation of ROS is disrupted, resulting in oxidant accumulation [13] and further inducing endoplasmic reticulum stress, DNA damage, mitochondrial dysfunction, and BCL-2/BAX apoptosis pathway activation [14]. Application of high doses of GCs induces severe oxidative stress by boosting the accumulation of intracellular ROS [15], thereby leading to apoptosis of BMSCs and further deterioration of the osteogenic and chondrogenic differentiation of BMSCs [16–18]. Oxidative stress also causes vascular endothelial dysfunction, resulting in blood vessel injury, which is another cause of SONFH pathogenesis [12]. Therefore, ROS activators have been used for establishing ONFH models in basic research [11], and reducing oxidative stress may constitute a therapeutic strategy for SONFH treatment.

Growth differentiation factor 15 (GDF15), also known as macrophage inhibitory cytokine-1, plays an important role in ROS generation and serves as a marker of oxidative stress in many diseases [19–21]. GDF15 is a member of the cell stress-responsive transforming growth factor *β* superfamily [22] and has been reported to suppress ROS generation via activation of the SMAD1 signaling pathway [23]. However, the specific role of GDF15 in bone metabolism is unclear. Westhrin et al. reported that GDF15 promotes osteoclast differentiation and inhibits osteoblast differentiation in multiple myeloma [24], whereas Symmank et al. demonstrated that mechanical stimulation can induce periodontal ligament cells to secrete more GDF15 and promote osteoblast differentiation [25]. Moreover, for several other types of cells, the AKT/mammalian target of rapamycin (mTOR) pathway acts downstream of GDF, and GDF15 could promote the survival and proliferation of cells via the activation of AKT/mTOR signaling pathway [26–28]. At the same time, the AKT/mTOR signaling pathway is closely associated with ROS, as its activation protects cells from ROS accumulation and restores the balance of ROS [29, 30]. Nonetheless, the mechanisms through which GDF15/AKT/mTOR signaling mediates the pathophysiological changes in association with SONFH and whether this signaling pathway can restore the redox balance in BMSCs under GC conditions are largely unknown.

In our previous study, we had screened cyclic polypeptide D7 from the cyclic peptide phage display library (Ph.D.-C7C) by phage display technology [31]. Phage display is a robust, high-throughput screening strategy in which a library of peptide or protein variants is expressed on the outside of a phage virion, and the genetic material encoding each variant is present on the inside of the virion [32]. Based on the different binding affinities of the cyclic peptide and the target cell, we screened D7 through three or four biopanning procedures, following previously described protocols [33, 34]. In addition, we demonstrated that D7 could enhance the adhesion, expansion, and proliferation of BMSCs on *β*-tricalcium phosphate scaffolds [31], in contrast to the pathophysiological changes related to SONFH.

Hence, in this study, we aimed to investigate the therapeutic effects of D7 on BMSCs proliferation and differentiation under SONFH conditions and elucidate the underlying mechanisms.

## 2. Methods and Materials

### 2.1. Reagents and Antibodies

Reagents for cell transfection were obtained from RIBOBIO (cat. no. C10511-05; Guangzhou, China). siRNA-GDF15 was synthesized by GEKYGENE (Shanghai, China). Reagents for cell differentiation and detection included ascorbic acid (cat. no. A8960-5G; Sigma-Aldrich, USA), dexamethasone (DEX; MCE, USA), paraformaldehyde (PFA), transforming growth factor (TGF)-*β*_1_ (cat. no. P01137; Peprotech, China), insulin, transferrin, and sodium selenite (ITS; cat. no. I3146; Sigma-Aldrich), and N-Acetyl-L-cysteine (NAC; cat. no. A9165; Sigma-Aldrich). Primary antibodies for Western blotting (WB) and immunofluorescence (IF) staining used in this study were from the following sources: anti-AKT (cat. no. 10176-2-AP), anti-phospho-AKT (cat. no. 66444-4-Ig), anti-glyceraldehyde 3-phosphate dehydrogenase (cat. no. 60004-1-Ig), anti-BCL-XL (cat. no. 10783-1-AP), BCL-2 (cat. no. 26593-1-AP), anti-BAX (cat. no. 50599-2-Ig), anti-poly (ADP-ribose) polymerase (PARP; cat. no. 66520-1-Ig), anti-aggrecan (cat. no. 13880-1-AP), anti-matrix metalloproteinase (MMP) 13 (cat. no. 18165-1-AP), anti-CD29 (cat. no. 12594-1-AP), anti-collagen type 2 (COL2; cat. no. 28459-1-AP), and anti-mTOR (cat. no. 66888-1-Ig) from Proteintech (China); anti-phospho-mTOR (cat. no. 5536S) from Cell Signaling Technology (Danvers, MA, USA); anti-GDF15 (cat. no. 27455-4-AP) from Santa Cruz Biotechnology (Dallas, TX, USA); and anti-SOX9 from Abcam (USA). Secondary antibodies for WB, including anti-mouse (cat. no. SA00001-1) and anti-rabbit (cat. no. SA00001-2) antibodies, were from Proteintech. Secondary antibodies for IF, including anti-mouse (cat. no. SA00003-1) and anti-rabbit (cat. no. SA00003-2) antibodies, were from Proteintech.

### 2.2. Synthesis of Peptides

The synthesis of peptides was performed as previously described [35]. The cyclic peptide (amino acid sequence CDNVAQSVC) contained seven amino acids; each amino acid terminus was conjugated with a pair of cysteine residues that formed an intramolecular disulfide linkage. All peptides were synthesized by solid-phase peptide synthesis using fluorenylmethyloxycarbonyl chemistry (Scilight-Peptide, Inc., Beijing, China).

### 2.3. Cell Culture

Sprague–Dawley rat BMSCs were purchased from Cyagen Biosciences, Inc. (cat. no. RASMX-01001; Guangzhou, China). BMSCs were grown at 37°C with a 5% CO_2_ atmosphere in complete medium (*α*-minimum essential medium (*α*-MEM) containing 10% fetal bovine serum (FBS) and 1% penicillin/streptomycin). The cells were passaged when they reached 70–80% confluence, and only passages 3–6 were used for experiments.

### 2.4. Western Blotting

WB was performed as previously described, with slight modifications [36]. Cells were lysed using ice-cold RIPA buffer containing 1% phenylmethylsulfonyl fluoride and 1% phosphatase inhibitor. Human tissue samples were added to RIPA lysis buffer at a ratio of 10 mg per 100 *μ*L. A tissue grinder was used to break the tissue. Protein concentrations were measured using BCA assays according to the manufacturer's instructions. Equal amounts of protein (20 *μ*g) were separated by sodium dodecyl sulfate polyacrylamide gel electrophoresis on 10% or 12% gels and transferred to polyvinylidene difluoride membranes. After blocking in TBST containing 5% fat-free milk, the membranes were incubated overnight at 4°C with primary antibodies. All primary antibodies were used at a dilution of 1 : 1000, except for anti-cleaved-caspase 3, which was used at a dilution of 1 : 500. The secondary antibody (diluted 1 : 5000) was incubated with membranes for 1 h at room temperature. Finally, the protein signals were visualized using Immobilon Western Chemiluminescent HRP Substrate (cat. no. WBKLS0500; Merck Millipore, Germany).

### 2.5. Reverse Transcription Quantitative Polymerase Chain Reaction (RT-qPCR)

RT-qPCR was performed as previously described [13]. We extracted total RNA using TRIzol reagent (TaKaRa Bio, China). One microgram RNA was used to synthesize cDNA using a PrimeScriptRT reagent Kit with gDNA Eraser (TaKaRa Bio). The sequences of the specific primers are shown in Table 1. PCR amplification was performed using a Roche LightCycler 480II (Roche, Basel, Switzerland). We analyzed the relative levels of gene expression using the 2^−△△CT^ method, with *β*-actin as an endogenous control.

### 2.6. Cell Viability Assay

Cell viability was evaluated using Cell Counting Kit-8 (CCK-8) assays (cat. no. HY-K0301; MCE, USA). Briefly, 5000 cells were seeded into each well of a 96-well plate and cultured in *α*-MEM containing 10% FBS. After treatment with DEX and other reagents for 72 h, the cells were washed thrice with phosphate-buffered saline (PBS). Then, 10 *μ*L CCK-8 solution in 100 *μ*L *α*-MEM was added to each well. After incubation for 2 h at 37°C in the dark, the absorbance was measured using a microplate reader at a wavelength of 450 nm.

### 2.7. Lactate Dehydrogenase (LDH) Release Assay

LDH release assays were performed as previously described [36]. Briefly, LDH was released from necrotic or apoptotic cells into the culture medium. BMSCs were seeded into 24-well plates and cultured in serum-free *α*-MEM with DEX and treated for 48 h. LDH activity was measured using a commercially available assay kit (Roche). The optical density was measured using a spectrophotometer at a wavelength of 490 nm.

### 2.8. EdU Incorporation Assay

Cell proliferation was evaluated by monitoring DNA synthesis using EdU incorporation assays. Briefly, 5000 cells/well were seeded into 96-well plates and incubated in *α*-MEM containing 10% FBS with specific treatments for 24 h. Next, EdU was diluted to 10 *μ*M and added to each well for 2 h. The EdU working solution provided in the kit (cat. no. C6015; Suzhou Yuheng Biotechnology, China) was mixed according to the manufacturer's instructions. Subsequently, the cells were fixed with 4% PFA for 20 min, permeabilized with 0.5% Triton X-100 in PBS for 5 min, blocked with 3% bovine serum albumin (BSA) for 1 h, and mixed with 100 *μ*L reagent according to the manufacturer's protocol.

### 2.9. Intracellular ROS Measurement

The level of intracellular ROS was evaluated using a reactive oxygen species assay kit (cat. no. S0033; Beyotime Biotechnology, Shanghai, China). Cells were seeded in six-well plates at a density of 30% and then transfected with siRNA for 24 h. DCFH-DA was then diluted in serum-free culture medium at a ratio of 1 : 1000 to a final concentration of 10 *μ*M. The cell culture medium was removed, and 1 mL diluted DCFH-DA was added to each well. After 30 min, DEX and D7 treatments were applied, and cells were again transfected with siRNA. Fluorescence intensity was observed by fluorescence microscopy after 30 min.

### 2.10. In Vitro Chondrogenic Induction of BMSCs

Chondrogenic differentiation of BMSCs was performed following a previously described method, with slight modifications [37]. BMSCs were plated into 12-well plates at a density of 2 × 10^5^ cells/well and incubated with *α*-MEM containing 10% FBS at 37°C and 5% CO_2_ for 24 h. After reaching 80% confluence, the cells were cultured in *α*-MEM containing 10% FBS, 1% penicillin-streptomycin, 1% ITS (Sigma-Aldrich), 100 nM DEX (MCE), 10 ng TGF-*β*1 (PeproTech), and 10 mg/mL ascorbic acid. Chondrogenic differentiation of BMSCs was induced for 21 days, during which time the medium was replaced every other day. The cells were then stained with Alcian blue.

### 2.11. Alcian Blue Staining

After incubation in chondrogenic inductive medium for 21 days, the cells were fixed in 4% PFA for 15 min. Thereafter, the cells were exposed to Alcian acidizing fluid (Solarbio, Beijing, China) for 3 min and Alcian staining solution for 30 min.

### 2.12. Immunofluorescence Staining

IF staining of cells was performed as previously described [38], and BMSCs were seeded on cover slips at an appropriate density (30–40% confluence). Twenty-four hours later, the cells were incubated with different treatments for 24 h. Cells were fixed with 4% PFA for 30 min and permeabilized with 0.5% Triton X-100 in PBS for 10 min. The cells were then blocked with 1% BSA for 1 h at room temperature. After incubation with primary antibody (anti-GDF15 diluted at 1 : 100) overnight at 4°C, the cells were incubated with the specific secondary antibody for 1 h and 4′,6-diamidino-2-phenylindole (DAPI; cat. no. C0065; Solarbio) for 5 min, sealed, and visualized using a Nikon fluorescence microscope (Axio Observer 3; Carl Zeiss AG, Germany).

After tissue isolation, human tissues were fixed in PFA for 48 h. Subsequently, tissues were decalcified in 10% ethylenediaminetetraacetic acid (EDTA)/PBS for 1 month. Decalcification was considered complete when a needle could easily penetrate the bone tissue. After embedding in paraffin, tissues were cut into 7 *μ*M-thick tissue sections for IF staining. After deparaffinization, the sample slices were used for IF staining. Briefly, the slices were placed in antigen repair solution, using a microwave oven at 100 W for 3 min to boil, followed by 50 W for 7 min. Heating was then stopped, and the samples were allowed to cool normally for 20–30 min. The slices were then permeabilized with 0.5% Triton-100X for 5 min and blocked with horse serum for 1 h at room temperature. After incubation with primary antibody (anti-CD29 diluted at 1 : 200; anti-GDF15 diluted at 1 : 100) overnight at 4°C, the slices were incubated with the specific secondary antibody for 1 h and DAPI (cat. no. C0065; Solarbio) for 5 min. Finally, the slices were incubated with Autofluo Quencher (cat. no. C1212; APPLYGEN, Beijing, China) for 10 min and covered with mounting medium, antifading (cat. no. S2100; Solarbio). The slices were sealed and visualized using a fluorescence microscope (Axio Observer 3; Carl Zeiss AG). All procedures involving human tissues were performed following protocols approved by the Shandong Provincial Hospital (Shandong, China; NSFC: No. 2019-187).

### 2.13. Terminal Deoxynucleotidyl Transferase dUTP Nick-End Labeling (TUNEL) Assay

Apoptotic cells were identified using a TUNEL staining kit (cat. no. KGA7072; KeyGEN BioTECH, China), as previously described [39]. Cells were seeded onto 24-well cover glasses and cultured in serum-free *α*-MEM with specific reagents for 48 h. The cells were then subjected to TUNEL staining according to the manufacturer's instructions, sealed, and visualized under a fluorescence microscope. Green staining indicated TUNEL-positive cells, and the percentage of positive cells was calculated in three random fields.

### 2.14. Flow Cytometry

The level of intracellular ROS was evaluated using a reactive oxygen species assay kit (cat. no. S0033; Beyotime Biotechnology, Shanghai, China). Cells were seeded in 6-well plates to reach 30% confluency and then transfected with siRNA for 24 h. DEX (10 *μ*M) and D7 (10 *μ*M) treatments were added into indicated culture medium. After 30 min, cells were digested with trypsin for 5 min. After stopping digestion with culture medium containing 10% FBS, cells were collected into 15 mL centrifuge tube, and centrifuged at 1500 rpm for 5 min. The cells were then resuspended with cold PBS and centrifuged again (1500 rpm for 5 min). Next, 1 mL diluted DCFH-DA solution (10 *μ*M) was added to each tube. Cells were cultured at 37°C in a 5% CO_2_ atmosphere for 30 min and shook every 5 min. Cells were then washed with PBS and centrifuged thrice. Thereafter, cells were resuspended in 400 *μ*L PBS and transferred to the flow cytometry test tube. The flow cytometry was used at the excitation wavelength of 488 nm and the emission wavelength of 525 nm to detect the fluorescence value.

### 2.15. Data Source

The dataset of GSE123568 included 40 samples, 10 of which were normal patient serum samples and 30 were SONFH serum samples. The data were deposited by Zhang et al. and downloaded from the GEO database (https://www.ncbi.nlm.nih.gov/geo/query/acc.cgi?acc=GSE123568) [40]. The datasets were obtained based on the GPL15207 platform, and the probe identification number was 11716663_a_at ([PrimeView] Affymetrix Human Gene Expression Array).

### 2.16. Statistical Analysis

All data are presented as means ± standard deviations (SDs; *n* > 3). *T*-tests were used for comparisons between two groups, and one-way analysis of variance was used for comparisons between more than two sets of data. Statistical significance was set at *p* < 0.05. All statistical analyses were performed with GraphPad Prism 8.0 software (GraphPad Software).

## 3. Results

### 3.1. GDF15 Expression is Suppressed in ONFH Samples

**Figure 1:**
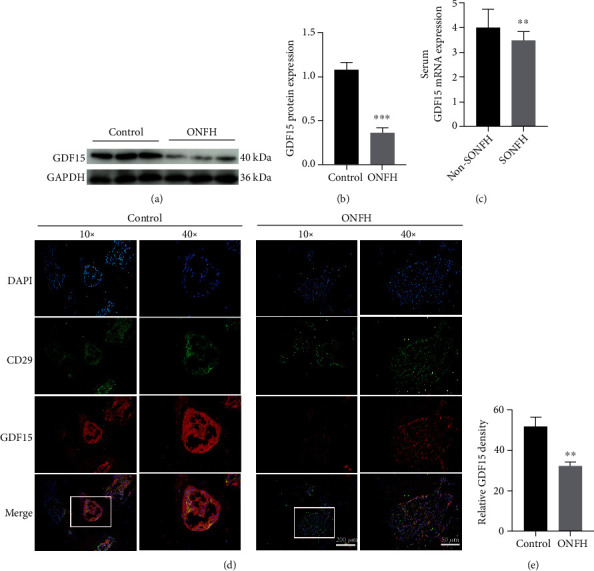
GDF15 expression is suppressed in ONFH samples. (a) Western blotting analysis of GDF15 expression in the ONFH and control groups (*n* = 3). (b) Quantitative analysis of the data from (a). ^∗∗∗^*p* < 0.01 versus the control group. (c) GDF15 expression in the GSE123568 dataset. (d) GDF15 expression in BMSCs from SONFH and healthy (control) femoral head tissues, as analyzed by immunofluorescence staining using anti-CD29 and GDF15 antibodies. (e) Quantitative analysis of the data from (d). ^∗∗^*p* < 0.01 versus the control group. Quantitative data are presented as means ± SD. ^∗∗^*p* < 0.01 versus the non-SONFH group.

To explore GDF15 expression levels during ONFH, we evaluated GDF15 expression in patients without ONFH (controls) and patients with ONFH. Using WB, we found that GDF15 protein levels were significantly downregulated in ONFH samples (Figure 1(a)). This result was further verified by data mining from the GEO dataset deposited by Zhang et al. (GSE123568; Figure 1(c)). The dataset included 40 serum samples, of which 10 were obtained from controls and 30 were obtained from patients with SONFH. As shown in Figure 1(c), GDF15 expression was markedly downregulated in the SONFH group. Additionally, we found that GDF15 expression in the BMSCs population was dramatically decreased under ONFH conditions (Figure 1(d)). Taken together, these results indicated that there was a negative correlation between GDF15 expression and ONFH and that BMSCs were one of the main cell populations affected in the bone microenvironment.

### 3.2. DEX Inhibits BMSCs Proliferation and Induces BMSCs Apoptosis via Suppression of GDF15 Expression and Downregulation of AKT/mTOR Signaling

**Figure 2:**
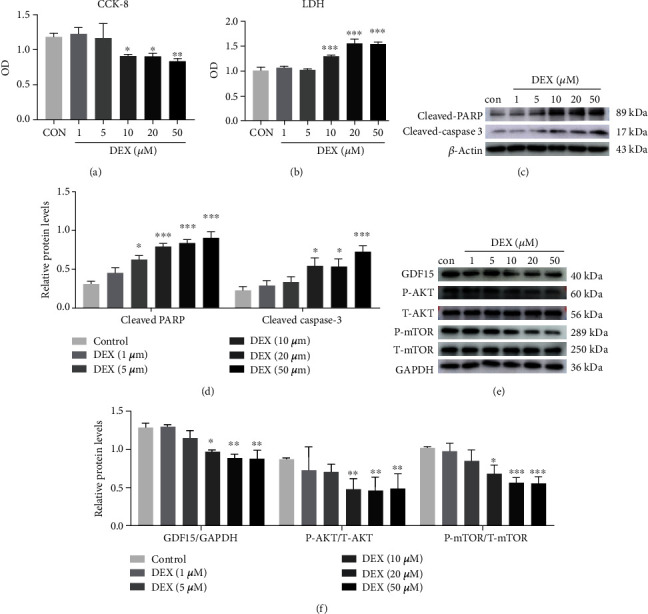
DEX inhibits BMSCs proliferation and induces BMSCs apoptosis via suppression of GDF15 expression and downregulation of AKT/mTOR signaling. (a) BMSCs viability following DEX stimulation, as evaluated by CCK-8 assays (*n* = 3). ^∗^*p* < 0.05, ^∗∗^*p* < 0.01 versus the control. Cells were incubated with complete medium for 2 days. (b) BMSCs apoptosis induced by DEX stimulation was measured by LDH release assays (*n* = 3). ^∗∗∗^*p* < 0.001 versus the control. Cells were treated with DEX in serum-free medium for 48 h. (c) Western blotting (WB) analysis of the effects of DEX (0–50 *μ*M) on the expression of apoptosis-related proteins (cleaved-PARP and cleaved-caspase 3) in BMSCs. Cells were treated with DEX in serum-free medium for 48 h before protein extraction. (d) Quantitative analysis of the data from (c) (*n* = 3). ^∗^*p* < 0.05, ^∗∗∗^*p* < 0.001 versus the control. (e) WB analysis of the effects of DEX (0–50 *μ*M) on GDF15 expression and AKT/mTOR phosphorylation in BMSCs. Cells were treated with DEX in complete medium for 48 h before protein extraction. (f) Quantitative analysis of the data from (e) (*n* = 3). ^∗^*p* < 0.05, ^∗∗^*p* < 0.01, ^∗∗∗^*p* < 0.001 versus the control. Quantitative data are presented as means ± SD.

To elucidate the functional roles of GDF15 and its downstream signaling in BMSCs during SONFH, DEX was used for a series of functional assays in vitro. Using CCK-8 assays, we found that BMSCs proliferation was inhibited by DEX stimulation when the concentration exceeded 10 *μ*M (Figure 2(a)). By contrast, BMSCs apoptosis was significantly enhanced by DEX stimulation, as measured by evaluation of LDH activity and cleaved-PARP and cleaved-caspase 3 levels (Figures 2(b)–2(d)). We further investigated whether GDF15 was involved in these DEX-dependent changes and evaluated the signaling pathways involved in this mechanism. As expected, WB results demonstrated relationships between GDF15 suppression and DEX stimulation (Figures 2(e) and 2(f)), wherein AKT/mTOR signaling was believed to be a downstream pathway. These results suggested a pivotal role of GDF15/AKT/mTOR signaling in DEX-induced damage to BMSCs self-renewal.

### 3.3. D7 Alleviates DEX-Induced BMSCs Damage in a Concentration-Dependent Manner

**Figure 3:**
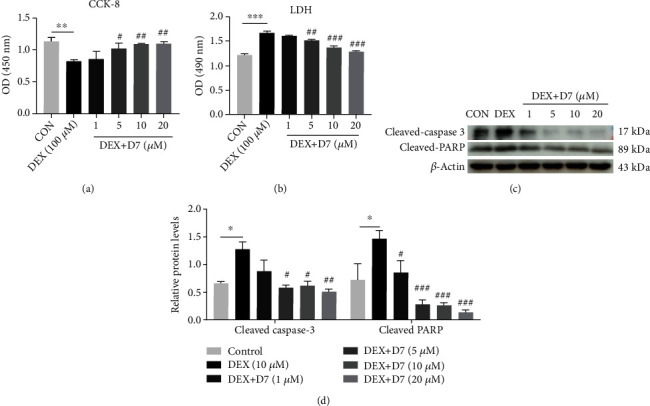
D7 alleviates DEX-induced BMSCs damage in a concentration-dependent manner. (a) The viability of BMSCs following DEX and D7 stimulation was examined by CCK-8 assays (*n* = 3). ^∗∗^*p* < 0.01 versus the control; ^#^*p* < 0.05, ^##^*p* < 0.01 versus DEX (10 *μ*M). Cells were incubated with complete medium for 2 days. (b) BMSCs apoptosis following DEX and D7 stimulation was examined by LDH release assays (*n* = 3). ^∗∗∗^*p* < 0.001 versus the control; ^##^*p* < 0.01, ^###^*p* < 0.001 versus DEX (10 *μ*M). Cells were treated with DEX and D7 in serum-free medium for 48 h before protein extraction. (c) WB analysis of the effects of D7 on cleaved-PARP and cleaved-caspase 3 levels in BMSCs (*n* = 3). ^∗∗∗^*p* < 0.001 versus the control; ^###^*p* < 0.001 versus DEX (10 *μ*M). Cells were treated with DEX and D7 in serum-free medium for 48 h before protein extraction. (d) Quantitative analysis of the data from (c) (*n* = 3). ^∗^*p* < 0.05 versus the control group; ^#^*p* < 0.05, ^##^*p* < 0.01, ^###^*p* < 0.001 versus DEX (10 *μ*M). Quantitative data are presented as means ± SD.

Our previous study demonstrated that the specific affinity cyclic peptide D7 could enhance the adhesion, expansion, and proliferation of BMSCs on *β*-tricalcium phosphate scaffolds [31]; therefore, we tested the potential therapeutic effects of D7 on DEX-induced BMSCs damage. To our surprise, D7 exhibited a powerful rescue effect against DEX-induced BMSCs damage, as demonstrated by concentration-dependent amelioration of BMSCs proliferation in CCK-8 assays (Figure 3(a)) and reduced expression of apoptosis markers, including LDH activity, cleaved-PARP levels, and cleaved-caspase 3 levels (Figures 3(b)–3(d)).

### 3.4. D7 Restores DEX-Dependent Inhibition of AKT/mTOR Signaling via Upregulation of GDF15 Expression in BMSCs

**Figure 4:**
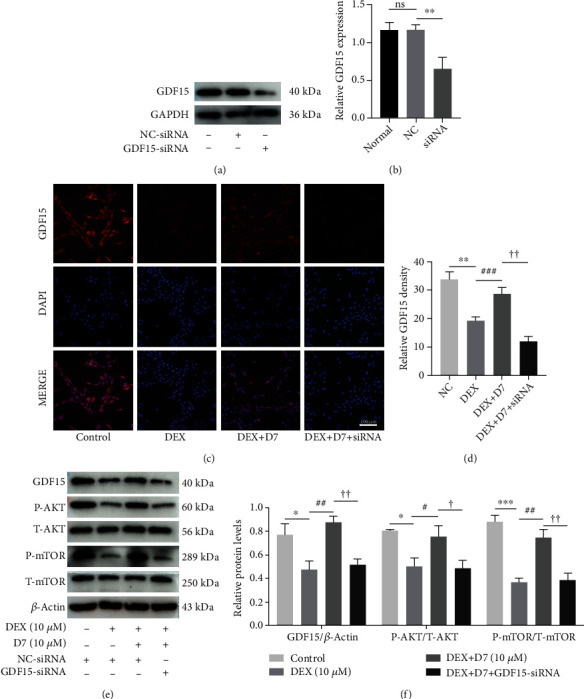
D7 restores DEX-dependent inhibition of AKT/mTOR signaling via upregulation of GDF15 expression in BMSCs. (a) Western blotting (WB) analysis of the efficiency of siRNA-GDF15 transfection. Cells were transfected with siRNA and incubated with complete medium for 48 h. (b) Quantitative analysis of data from (a) (*n* = 3). ^∗∗^*p* < 0.01 versus the normal and negative control groups. (c) Immunofluorescence analysis of GDF15 expression in BMSCs under different experimental conditions. Cells were transfected with siRNA and treated with different reagents for 24 h. (d) Quantitative analysis of data from (c) (*n* = 3). ^∗∗^*p* < 0.01 versus the NC group; ^###^*p* < 0.001 versus the DEX group; ^††^*p* < 0.01 versus the DEX + D7 group. (e) WB analysis of GDF15 expression and AKT/mTOR phosphorylation in BMSCs under different experimental conditions. (f) Quantitative analysis of data from (e) (*n* = 3). ^∗^*p* < 0.05, ^∗∗∗^*p* < 0.001 versus the control group; ^#^*p* < 0.05, ^##^*p* < 0.01 versus the DEX group; ^†^*p* < 0.05, ^††^*p* < 0.01 versus the DEX + D7 group. Quantitative data are presented as means ± SD.

Considering the pivotal role of GDF15/AKT/mTOR signaling in DEX-induced BMSCs damage, we next investigated whether this signaling pathway was also involved in the therapeutic effects of D7. siRNA-GDF15 was introduced for further studies, and the efficiency of the siRNA effect was evaluated by WB (Figures 4(a) and 4(b)). Consistent with our previous results, IF staining demonstrated that GDF15 expression was suppressed in the presence of DEX stimulation, whereas D7 restored GDF15 activation (Figures 4(c) and 4(d)). In addition, transfection with GDF15-siRNA strongly blocked D7-dependent restoration of GDF15 expression, indicating a regulatory role for GDF15 in mediating the therapeutic effects of D7 against DEX-induced BMSCs damage (Figures 4(c) and 4(d)). This mechanism was further supported by the WB results, wherein the DEX-dependent inhibition of AKT/mTOR activity was restored by D7 via GDF15 upregulation and then partially blocked after GDF15-siRNA transfection (Figures 4(e) and 4(f)). These findings indicated the downstream roles of the AKT/mTOR signaling pathway in the therapeutic effects of D7 against DEX-induced BMSCs damage.

### 3.5. D7 Reduces DEX-Induced Oxidative Stress via Regulation of GDF15 Expression

**Figure 5:**
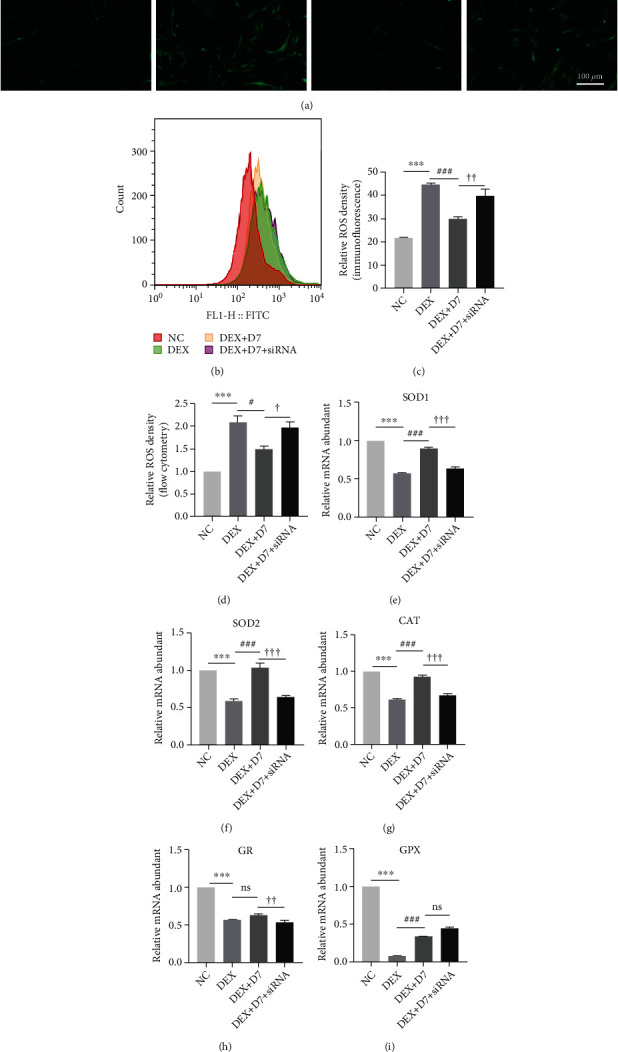
D7 reduces DEX-induced oxidative stress via regulation of GDF15 expression. (a) DCFH-DA assays were used to evaluate intracellular ROS levels in BMSCs under different experimental conditions. Cells were loaded with DCFH-DA for 30 min and treated with different reagents for 30 min. (b) The density of DCFH-DA was detected by flow cytometry. Cells were loaded with DCFH-DA for 30 min and treated with different reagents for 30 min. (c) Quantitative analysis of data from (a) (*n* = 3). ^∗∗∗^*p* < 0.001 versus the NC group; ^###^*p* < 0.001 versus the DEX group; ^†††^*p* < 0.001 versus the DEX + D7 group. (d) Quantitative analysis of data from (b) (*n* = 3). ^∗∗∗^*p* < 0.001 versus the NC group; ^#^*p* < 0.05 versus the DEX group; ^†^*p* < 0.05 versus the DEX + D7 group. (e–i) mRNA levels of *SOD1*, *SOD2*, *CAT*, *GR*, and *GPX* under different experimental conditions, normalized to *ACTB* expression (*n* = 3). Cells were treated with different reagents for 24 h. Quantitative data are presented as means ± SD.

GDF15 serves as a marker of oxidative stress and inflammation in many diseases and pathophysiological assessments [19–21]. Therefore, we next tested whether GDF15 had similar effects on the therapeutic action of D7 against DEX-induced BMSCs damage and whether ROS functioned upstream of GDF15 during this process because DEX enhances ROS levels and induces cell death in chondrocytes [41, 42]. Using the DCFH-DA probe, we found that intracellular ROS levels increased dramatically in DEX-stimulated BMSCs and decreased rapidly after D7 addition (Figures 5(a) and 5(c)). These results were further verified by DCFH-DA flow cytometry analysis (Figures 5(b) and 5(d)). However, when GDF15 expression was inhibited by siRNA, D7 partially reduced DEX-induced ROS production in BMSCs (Figures 5(a) and 5(c)). Then, we used N-acetyl-l-cysteine (NAC) to suppress DEX-induced oxidative stress to verify that ROS functioned upstream of GDF15/AKT/mTOR signaling. As expected, the activation of GDF15/AKT/mTOR signaling suppressed by DEX was restored by NAC treatment (Supplement Figure 1a and 1b).

We then evaluated the responses of antioxidant proteins to determine whether the defense system of oxidative stress contributed to the GDF15-mediated therapeutic effects of D7. qPCR showed that DEX reduced the expression of all five antioxidant genes. However, D7 only restored the expression of superoxide dismutase (*SOD*) 1, *SOD2*, and catalase (*CAT*) via a mechanism involving GDF15 (Figures 5(e)–5(g)). Glutathione reductase (*GR*) and glutathione peroxidase (*GPX*) were either not responsive to D7 treatment or were not regulated by GDF15 (Figures 5(h) and 5(i)). Taken together, these results suggested that D7 reduced DEX-induced ROS activity and restored the redox balance via regulation of GDF15 expression.

### 3.6. D7 Restores BMSCs Viability and Reduces BMSCs Apoptosis via GDF15 Expression

**Figure 6:**
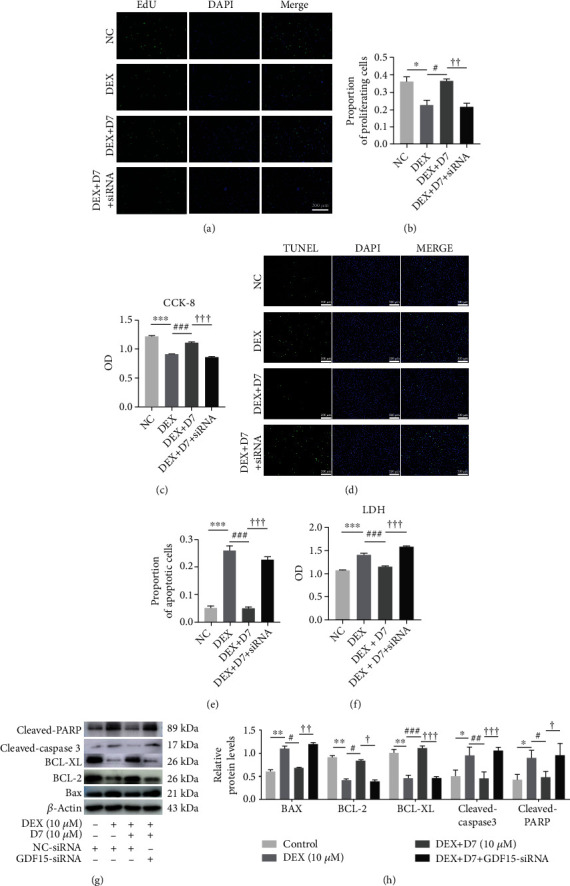
D7 restores BMSCs viability and reduces BMSCs apoptosis via GDF15 expression. (a) EdU assays were performed to evaluate the proportion of proliferating BMSCs under different experimental conditions. Cells were treated with different reagents for 24 h and were then incubated with EdU solution for 2 h. (b) Quantitative analysis of data from (a) (*n* = 3). ^∗^*p* < 0.05 versus the NC group; ^#^*p* < 0.05 versus the DEX group; ^††^*p* < 0.01 versus the DEX + D7 group. (c) Viability of BMSCs under different conditions was examined by CCK-8 assays (*n* = 3). ^∗∗∗^*p* < 0.001 versus the NC group; ^###^*p* < 0.001 versus the DEX group; ^†††^*p* < 0.001 versus the DEX + D7 group. (d) TUNEL assays were performed to measure the proportion of apoptotic BMSCs under different conditions. Cells were incubated in serum-free medium for 48 h before detection. (e) Quantitative analysis of data from (d) (*n* = 3). ^∗∗∗^*p* < 0.001 versus the NC group; ^###^*p* < 0.001 versus the DEX group; ^†††^*p* < 0.001 versus the DEX + D7 group. (f) Apoptosis of BMSCs under different condition was measured by LDH assays (*n* = 3). Cells were incubated in serum-free medium for 48 h before detection. ^∗∗∗^*p* < 0.001 versus the NC group; ^###^*p* < 0.001 versus the DEX group; ^†††^*p* < 0.001 versus the DEX + D7 group. (g) Western blotting analysis of the expression of some apoptosis-related proteins in BMSCs under different experimental conditions. Cells were incubated in serum-free medium for 48 h before lysis. (h) Quantitative analysis of data from (g) (*n* = 3). ^∗^*p* < 0.05, ^∗∗^*p* < 0.01 versus the NC group; ^#^*p* < 0.05, ^##^*p* < 0.01, ^###^*p* < 0.001 versus the DEX group; ^†^*p* < 0.05, ^††^*p* < 0.01, ^†††^*p* < 0.001 versus the DEX + D7 group. Quantitative data are presented as means ± SD.

We next determined whether GDF15-mediated signaling affected the therapeutic action of D7 against DEX-induced BMSCs damage. As expected, the proliferation of BMSCs damaged by DEX treatment was restored after D7 addition in the presence of GDF15, as demonstrated by EdU and CCK-8 assays (Figures 6(a)–6(c)) Moreover, apoptosis indices, as evaluated by TUNEL assays and LDH release tests, were all reduced (Figures 6(d)–6(f)). These results were further validated at the protein level; the expression levels of apoptosis-related proteins, including cleaved-PARP and cleaved-caspase 3, were decreased, whereas those of antiapoptotic proteins, including BCL-2 and BCL-XL, were increased (Figures 6(g) and 6(h)), supporting the involvement of GDF15-mediated signaling in the therapeutic effects of D7.

### 3.7. D7 Promotes Chondrogenesis following DEX Stimulation via GDF15 Expression

**Figure 7:**
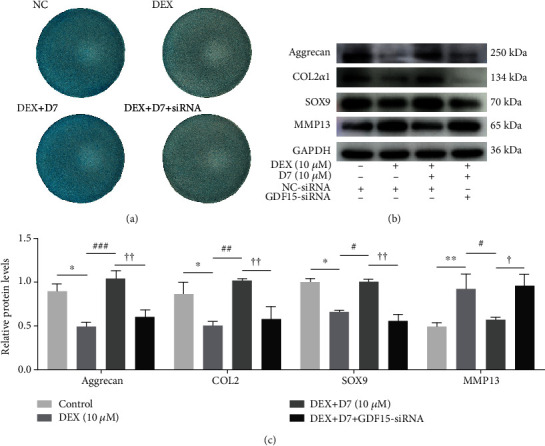
D7 promotes chondrogenesis following DEX stimulation via GDF15 expression. (a) Representative images of Alcian blue staining. Chondrogenic differentiation was induced in BMSCs for 21 days. (b) WB analysis of the expression of some chondrogenesis-related proteins, including Aggrecan, SOX9, COL2, and MMP13. Chondrogenesis was induced for 14 days. (c) Quantitative analysis of data from (b) (*n* = 3). ^∗^*p* < 0.05, ^∗∗^*p* < 0.01 versus the NC group; ^#^*p* < 0.05, ^##^*p* < 0.03, ^###^*p* < 0.001 versus DEX (10 *μ*M); ^†^*p* < 0.05, ^††^*p* < 0.01 versus the DEX + D7 group. Quantitative data are presented as means ± SD.

Because of the therapeutic effects of D7 on DEX-induced BMSCs damage, we next evaluated whether the chondrogenic differentiation of BMSCs under DEX conditions could benefit from D7 addition. As expected, D7 promoted the chondrogenic differentiation of DEX-treated BMSCs, and the effect of D7 was partially blocked by GDF15-siRNA transfection (Figure 7(a)). These results were further verified by WB analysis of protein expression. Notably, D7 increased the expression levels of Aggrecan (ACAN), COL2, and SOX9 and reduced the expression of MMP13. However, GDF15-siRNA transfection significantly blocked the D7-enhanced expression of chondrogenesis-related proteins, indicating a regulatory role of GDF15 during D7-induced chondrogenic differentiation in the presence of DEX (Figures 7(b) and 7(c)).

## 4. Discussion

**Figure 8:**
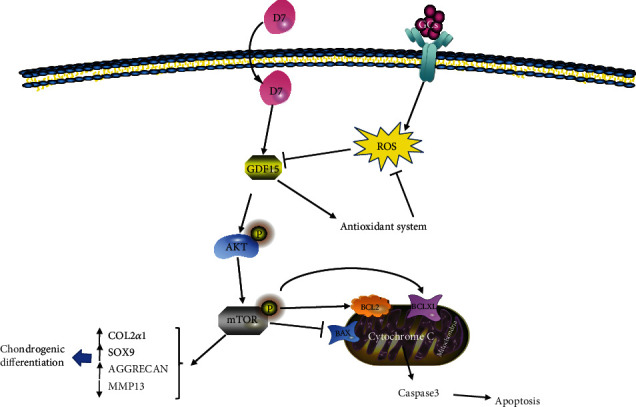
Schematic diagram showing the underlying mechanism through which D7 prevents SONFH. Glucocorticoids lead to the accumulation of ROS in BMSCs, thereby inhibiting GDF15/AKT/mTOR signaling and further causing damage to BMSCs. D7 restores the expression of GDF15, activates the oxidative stress defense system, and restores the balance of oxidative stress. GDF15 further activates the AKT/mTOR signaling pathway to protect BMSCs from SONFH.

In our previous study, we had demonstrated that the cyclic polypeptide D7 has a specific affinity to BMSCs [35]. To the best of our knowledge, our study is the first to demonstrate that D7 protected BMSCs against DEX-induced SONFH in vitro. In the current study, we found that D7 promoted the viability of BMSCs in the presence of DEX and reduced DEX-induced cell apoptosis. Activation of the GDF15/AKT/mTOR signaling pathway contributed to the protective roles of D7 in the DEX-induced SONFH model. Furthermore, activation of GDF15/AKT/mTOR signaling contributed to the suppressive effects of D7 on oxidative stress, mediated by restoration of SOD1, SOD2, and CAT expression. Additionally, D7 promoted BMSCs chondrogenesis through GDF15/AKT/mTOR signaling during SONFH (Figure 8). These results established the potential therapeutic effects of D7 in SONFH for the first time.

GCs are a common cause of ONFH as well as many other bone diseases [43, 44]. By inducing the accumulation of ROS, DEX promotes osteoblast apoptosis, inhibits the osteoblastic differentiation of BMSCs, and activates osteoclasts [16–18]. In this study, we also found that DEX could cause severe damage to BMSCs, including inhibition of cell proliferation and acceleration of cell apoptosis, via the regulation of BCL-2 family and caspase3 expression, and the suppression on GDF15/AKT/mTOR signaling. The chondrogenesis of BMSCs was therefore significantly inhibited by DEX treatment. Moreover, we found that DEX activated intracellular ROS by inhibiting the expression of the antioxidant proteins SOD1, SOD2, GR, GPX1, and CAT.

In our previous study, we screened a BMSCs-specific affinity peptide, D7, using phage display technology [35]. Similar peptides, such as RGD, have been used in the treatment of ONFH and have shown some therapeutic effects [45, 46]. Compared with RGD, D7 has specific affinity for BMSCs and the ability to recruit BMSCs more effectively. In this study, we showed that D7 treatment could alleviate the BMSCs injury caused by DEX. Furthermore, D7 was found to restore the chondrogenic function of BMSCs. In addition, D7 reestablished the balance of oxidative stress by regulating the expression of SOD1, SOD2, and CAT and exerted protective effects by activating GDF15/AKT/mTOR signaling.

Deregulation of the expression and function of cytokines belonging to the TGF-*β* family always contribute to the apoptosis and oxidative. Apoptosis is one of the important forms of cell death, and it plays an important role in the pathogenesis and progression of femoral head necrosis [47]. The BCL-2 family can regulate cell apoptosis. BAX, BCL-2, and BCL-XL are important regulators belonging to the BCL-2 family that control apoptosis. BAX can inhibit the activity of BCL-XL, while BCL-2 can inhibit BAX expression. Caspase family also plays an important role in the process of apoptosis. BCL-2 can inhibit the conversion of cytochrome C into caspase3, thereby inhibiting apoptosis. In this study, we showed that GDF15 expression was suppressed in femoral head tissue from patients with ONFH. Consistent with this, we also found that GDF15 expression was lower in the serum of patients with SONFH than in healthy controls. Importantly, DEX decreased GDF15 expression and inhibited the AKT/mTOR signaling pathway. D7 could upregulate the expression of BCL-2 and BCL-XL by activating GDF15/AKT/mTOR signaling and suppressed the expression of BAX, which further inhibited the formation of caspase3 and further alleviated cell apoptosis. For oxidative stress, GC can mediate the accumulation of oxygen free radicals by inhibiting the expression of antioxidant proteins. In this study, we also found GC inhibited the expression of SOD1, SOD2, GR, GPX1, and CAT to activate oxidative stress. According to other reports, GDF15/AKT/mTOR signaling is closely related to oxidative stress, wherein the activation of this signaling could suppress ROS accumulation [19–21, 23, 29, 48, 49]. In our study, D7 treatment was observed to inhibit the GC-induced oxidative stress by restoring the expression levels of SOD1, SOD2, CAT, and GPX. The silencing of GDF15 significantly reverses the antioxidant effect of D7 by downregulating the expression of SOD1, SOD2, and CAT. In general, GDF15/AKT/mTOR signal inhibited GC-induced oxidative stress by upregulating the expression of SOD1, SOD2, and CAT. Furthermore, as a member of the TGF-*β* superfamily, GDF15 is closely related to the differentiation of BMSCs [25] and contributes to the pathological progression of SONFH. In our study, D7 treatment could restore the chondrogenic differentiation function of BMSCs, whereas siRNA-GDF15 transfection inhibited the D7-induced chondrogenic differentiation of BMSCs.

Overall, our findings clarified the interactions among D7, GDF15, oxidative stress, and SONFH. However, further in vivo experiments are needed to clarify the therapeutic effects of D7-containing scaffolds in animal models of SONFH.

In conclusion, we demonstrated the protective effects of D7 against DEX-induced SONFH in vitro and found that these protective effects may be mediated by inhibition of DEX-induced ROS and activation of GDF15/AKT/mTOR signaling. Our research provides insights into the potential applications of D7 in the treatment of SONFH.

## Figures and Tables

**Table 1:** Primer sequences for quantitative real-time PCR.

Gene	Forward primer	Reverse primer
*ACTB*	GGAGATTACTGCCCTGGCTCCTA	GACTCATCGTACTCCTGCTTGCTG
*SOD1*	GGCAAAGGTGGAAATGAAGAAA	CAGTTTAGCAGGACAGCAGATGAG
*SOD2*	CATACTTGGTGTGAGCTGCTCTTGA	TTTGATGGCCTTATGATGACAGTGA
*GPX1*	AGGAGAATGGCAAGAATGAAGAGA	GGAAGGTAAAGAGCGGGTGAG
*GR*	TGCCCTGGGTTGGAGATCATA	TGGTCATACATGCAGGGTAGAGACA
*CAT*	GAACATTGCCAACCACCTGAAAG	GTAGTCAGGGTGGACGTCAGTGAA

*ACTB*: *β*-Actin; *SOD1*: superoxide dismutase 1; *SOD2*: superoxide dismutase 2; *GPX1*: glutathione peroxidase; *GR*: glutathione reductase; *CAT*: catalase.

## Data Availability

The data will be made available after being requested from the corresponding author.

## Supplementary Materials

Supplementary Materials Supplemental Figure 1: NAC restores DEX-dependent inhibition of GDF15/AKT/mTOR signaling. (a) WB analysis of GDF15 expression and AKT/mTOR phosphorylation in BMSCs under DEX and NAC treatment. (b) Quantitative analysis of data from (a). ^∗^*p* < 0.05 versus the NC group; ^#^*p* < 0.05 versus the DEX group.
