# Supplementary figures and images for: Cyclic Polypeptide D7 Protects Bone Marrow Mesenchymal Cells and Promotes Chondrogenesis during Osteonecrosis of the Femoral Head via Growth Differentiation Factor 15-Mediated Redox Signaling
Source: Oxid Med Cell Longev. 2022 Mar 3;2022:3182368. doi: 10.1155/2022/3182368 (PMC8913072; doi:10.1155/2022/3182368)

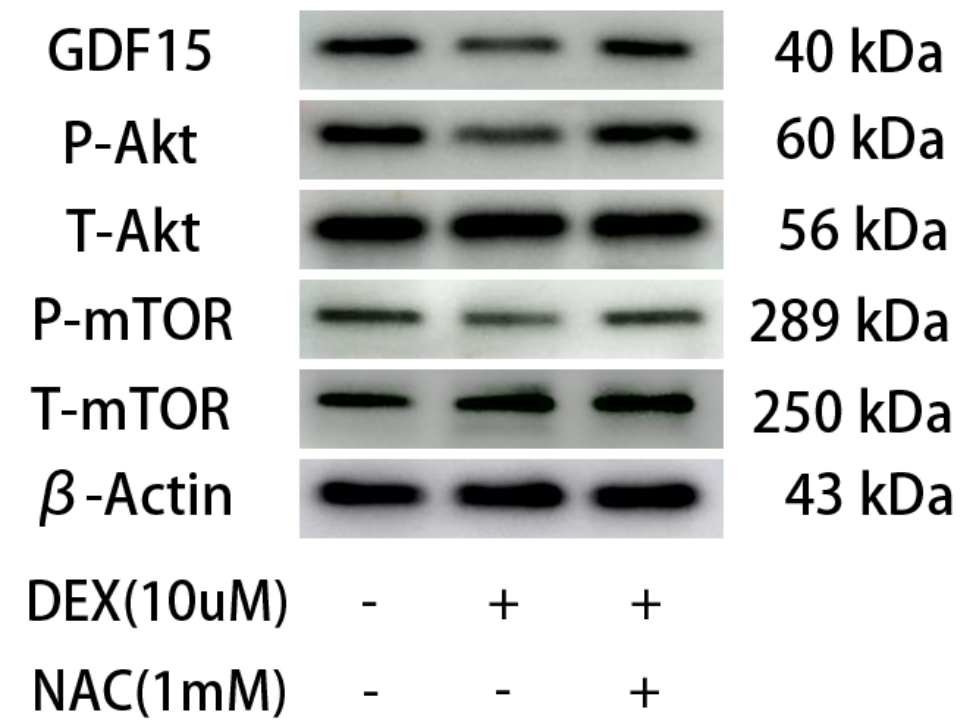

(a)

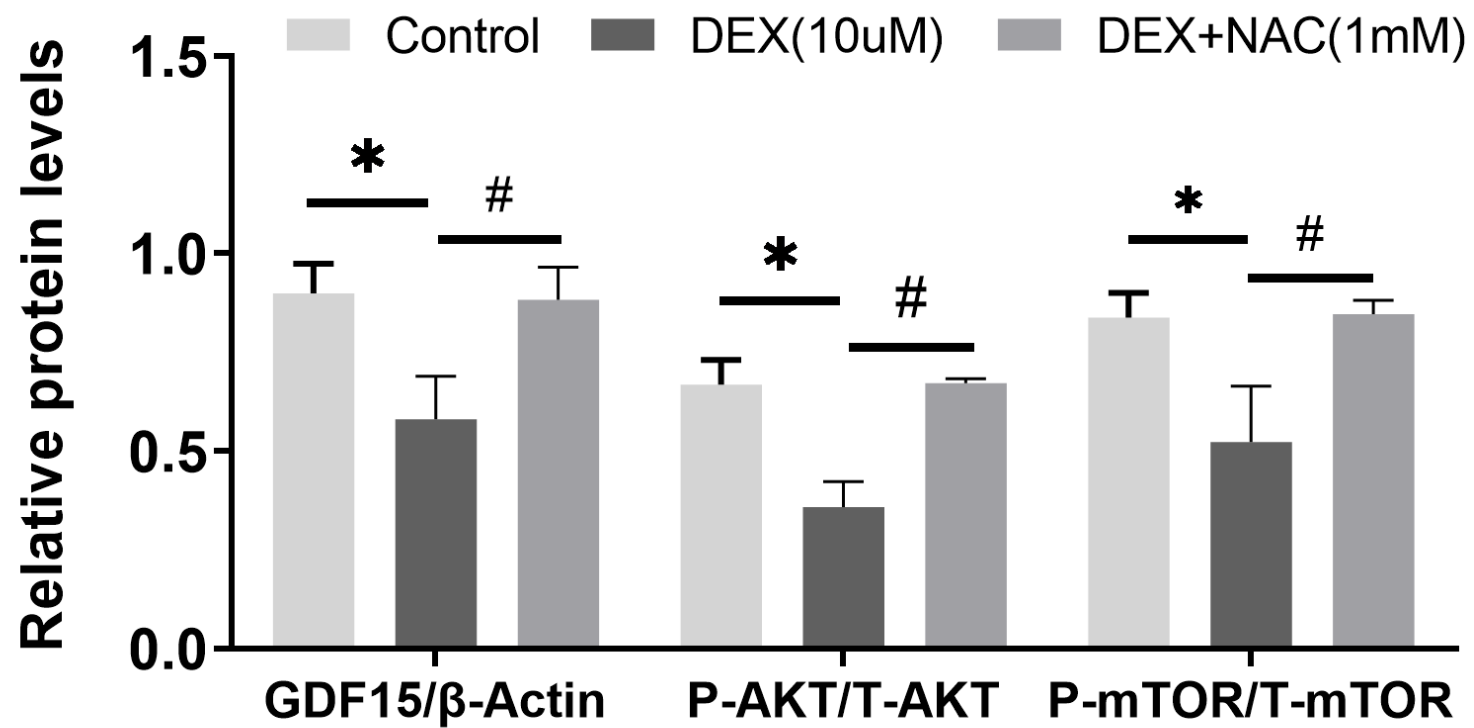

(b)

Supplement: Supplementary Materials — Supplemental Figure 1: NAC restores DEX-dependent inhibition of GDF15/AKT/mTOR signaling. (a) WB analysis of GDF15 expression and AKT/mTOR phosphorylation in BMSCs under DEX and NAC treatment. (b) Quantitative analysis of data from (a). ∗p < 0.05 versus the NC group; #p < 0.05 versus the DEX group. [file 3182368.f1.pdf]
